# Early patient and liver allograft outcomes from donation after circulatory death donors using thoracoabdominal normothermic regional: a multi-center observational experience

**DOI:** 10.3389/frtra.2023.1184620

**Published:** 2023-09-11

**Authors:** Aleah L. Brubaker, Raeda Taj, Brandon Jackson, Arielle Lee, Catherine Tsai, Jennifer Berumen, Justin R. Parekh, Kristin L. Mekeel, Alexander R. Gupta, James M. Gardner, Thomas Chaly, Amit K. Mathur, Caroline Jadlowiec, Sudhakar Reddy, Rafael Nunez, Janet Bellingham, Elizabeth M. Thomas, Jason R. Wellen, Jenny H. Pan, Mark Kearns, Victor Pretorius, Gabriel T. Schnickel

**Affiliations:** ^1^ Department of Surgery, Division of Transplant and Hepatobiliary Surgery, UC San Diego, San Diego, CA, United States; ^2^ Department of Surgery, Division of Cardiothoracic Surgery, UC San Diego, San Diego, CA, United States; ^3^ Department of Surgery, Division of Transplant Surgery, UC San Francisco, San Francisco, CA, United States; ^4^ Arizona Transplant Associates, Phoenix, AZ, United States; ^5^ Department of Surgery, Division of Transplant Surgery, Mayo Clinic Arizona, Phoenix, AZ, United States; ^6^ Department of Transplantation, California Pacific Medical Center, San Francisco, CA, United States; ^7^ Department of Surgery, University Transplant Center, University of Texas Health San Antonio, San Antonio, TX, United States; ^8^ Department of Surgery, Section of Abdominal Transplantation, Washington University School of Medicine, St Louis, MO, United States; ^9^ Department of Surgery, Division of Abdominal Transplantation, Stanford University, Stanford, CA, United States

**Keywords:** thoracoabdominal normothermic regional perfusion (TA-NRP), donation after circulatory death, liver transplant, organ procurement, transplant outcomes

## Abstract

**Background:**

Donation after circulatory death (DCD) liver allografts are associated with higher rates of primary non-function (PNF) and ischemic cholangiopathy (IC). Advanced recovery techniques, including thoracoabdominal normothermic regional perfusion (TA-NRP), may improve organ utilization and patient and allograft outcomes. Given the increasing US experience with TA-NRP DCD recovery, we evaluated outcomes of DCD liver allografts transplanted after TA-NRP.

**Methods:**

Liver allografts transplanted from DCD donors after TA-NRP were identified from 5/1/2021 to 1/31/2022 across 8 centers. Donor data included demographics, functional warm ischemic time (fWIT), total warm ischemia time (tWIT) and total time on TA-NRP. Recipient data included demographics, model of end stage liver disease (MELD) score, etiology of liver disease, PNF, cold ischemic time (CIT), liver function tests, intensive care unit (ICU) and hospital length of stay (LOS), post-operative transplant related complications.

**Results:**

The donors' median age was 32 years old and median BMI was 27.4. Median fWIT was 20.5 min; fWIT exceeded 30 min in two donors. Median time to initiation of TA-NRP was 4 min and median time on bypass was 66 min. The median recipient listed MELD and MELD at transplant were 22 and 21, respectively. Median allograft CIT was 292 min. The median length of follow up was 257 days. Median ICU and hospital LOS were 2 and 7 days, respectively. Three recipients required management of anastomotic biliary strictures. No patients demonstrated IC, PNF or required re-transplantation.

**Conclusion:**

Liver allografts from TA-NRP DCD donors demonstrated good early allograft and recipient outcomes.

## Introduction

Maximizing safe utilization of extended criteria donors is required to meet the ongoing organ shortage for liver transplantation. While interest in and utilization of allografts from donation after circulatory death (DCD) donors has grown, liver allografts from DCD donors are associated with higher rates of primary nonfunction (PNF), early allograft dysfunction (EAD), and ischemic cholangiopathy (IC) owing to warm ischemia (1–3).

Given the associated complications with DCD donors, the United States (US) has been slow to adopt broader consideration of DCD donors and has a relatively low rate of DCD liver allograft utilization. In the US, DCD donors made up 16.9% of the donor pool in 2016; this number has risen to over 25% in 2020 and 2021 (4). Despite the growth of the DCD donor pool, the rate of DCD liver transplantation has not kept pace. In the United Kingdom (UK) at least 1/5 of liver transplants are from DCD donors, while livers transplanted from DCD donors in the US accounted for between 6 and 12% between 2016 and 2020 (4, 5).

Normothermic regional perfusion (NRP) optimizes DCD donors via *in situ* machine perfusion of oxygenated blood to targeted organs after declaration of cardiopulmonary arrest. In Europe, abdominal NRP with either pre- or post-mortem cannulation has demonstrated excellent liver allograft outcomes. Compared to matched cohorts of DBD donors, NRP DCD donors had similar rates of EAD, IC, need for re-transplantation, and 1–3 year graft survival (6–12).

The DCD transplant landscape is being disrupted by a variety of novel technologies including *in situ* NRP, *ex situ* normothermic machine perfusion (NMP) and *ex situ* hypothermic machine perfusion. With the growing use of DCD donors for heart transplant, cardiac transplant surgeons have successfully used thoracoabdominal normothermic regional perfusion (TA-NRP) and *ex situ* NMP to increase the donor pool and access to cardiac transplant (13, 14). Similary *ex situ* NMP and HMP for liver allografts has demonstrated promising results for maringal and DCD liver allografts (15–17). While prior publications from our European colleagues have evaluated the use of abdominal NRP for liver allografts, the data in respect to TA-NRP has been more limited. A recent publication by Sellers et al. supported the European data and demonstrated good liver allograft outcomes after TA-NRP across several US transplant centers (18).

At University of California San Diego (UCSD), the cardiac transplant team has successfully performed TA-NRP for 30 donors utilizing a modified cardiopulmonary bypass (CPB) circuit over our study period. Liver allografts were transplanted in just over half of these cases. Given the increasing experience of DCD TA-NRP for abdominal allografts, we sought to evaluate early outcomes of DCD liver allografts transplanted after TA-NRP and add to the growing US literature in support of TA-NRP as a recovery modality that has the potential to increase DCD organ utilization.

## Methods

### Study population

**Figure 1:**
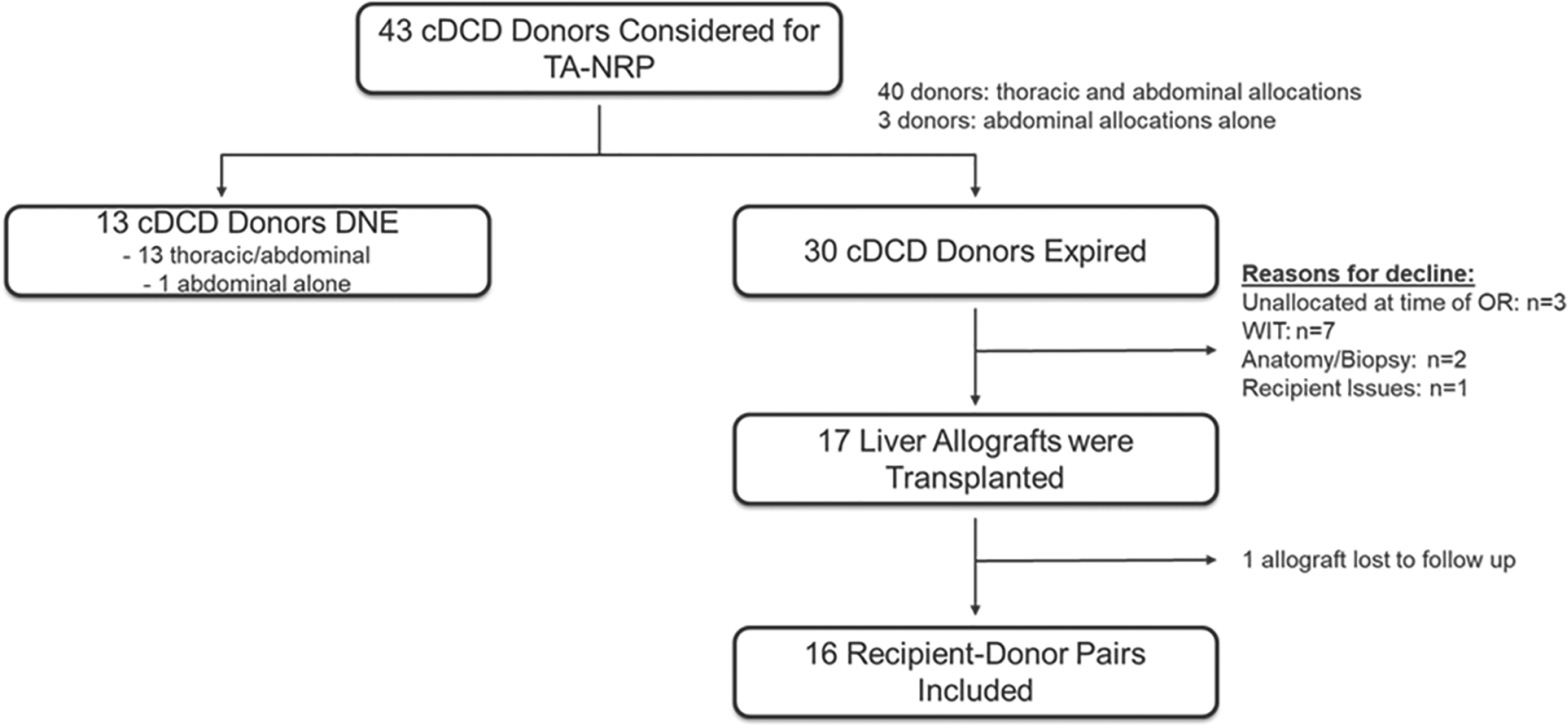
Retrospective observational study design. Flow diagram representing the number of potential donors evaluated for TA-NRP, donors that expired, number of liver allografts transplanted and number of allograft:recipient pairs included in the study.

Use of TA-NRP for DCD by the UCSD cardiac transplant team began in May 2021. From May 1, 2021 thru January 31, 2022, a total of 43 DCD donors were considered for TA-NRP procurement; 40 donors were considered for thoracic and abdominal transplantation and 3 donors for abdominal transplantation alone at the time of withdrawal of life sustaining therapy (WLST). Characteristics of all 43 donors considered for TA-NRP are in Supplementary Table S1. 13 donors did not expire within 90 min after extubation. Of the 30 TA-NRP DCD donors that expired, 17 liver allografts were transplanted. 16 liver allografts and recipients were retrospectively identified at multiple transplant centers (*n* = 8); one center was unable to be reached for follow-up data (Figure 1). The principal inclusion criteria was acceptance and transplant of a liver allograft after a DCD donor underwent TA-NRP performed by the UCSD NRP team. There was no exclusion criteria. Liver allograft acceptance for each recipient was at the discretion of the accepting center's standard practice for liver transplantation.

TA-NRP was conducted under two clinical scenarios. Most commonly, TA-NRP was conducted in DCD donors to assess and procure thoracic and abdominal organs for transplant. In three cases, TA-NRP was considered in DCD donors solely for the assessment and procurement of abdominal organs; two of these donors expired. Supplementary Table S2 demonstrates which organs were allocated, accepted for transplant, recovered for research or discarded. Ultimately, 25 hearts, 5 lungs, 17 livers, 54 kidneys and 4 pancreata were transplanted. Additional evaluation of other allograft outcomes after TA-NRP will be the target of future studies.

De-identified recipient data were collected locally at each transplant center with existing institutional review board (IRB) approval. UCSD provided an IRB exemption for the analysis of pooled anonymous data. Donor cause of death, demographics, laboratory data, functional warm ischemia time (fWIT and total warm ischemia time (tWIT) were obtained through the United Network for Organ Sharing (UNOS). TA-NRP recovery data, including time from incision to bypass initiation and total time on bypass, were recorded and stored at UCSD. Recipient data include age, sex, model of end stage liver disease (MELD) score, etiology of liver disease, primary non-function (PNF), cold ischemic time (CIT), post-reperfusion peak aspartate aminotransferase (AST, U/L), alanine transaminase (ALT, U/L) and lactate (mmol/L), intensive care unit (ICU) length of stay (LOS), hospital LOS, post-operative transplant related complications and transplant related readmissions to date. Length of follow up was sensored at the last date of data provided for a given patient. The UK DCD risk score was retrospectively calculated for all donor-recipient pairs (19). All aggregate data are presented as the median with interquartile range (IQR).

### TA-NRP and DCD

**Figure 2:**
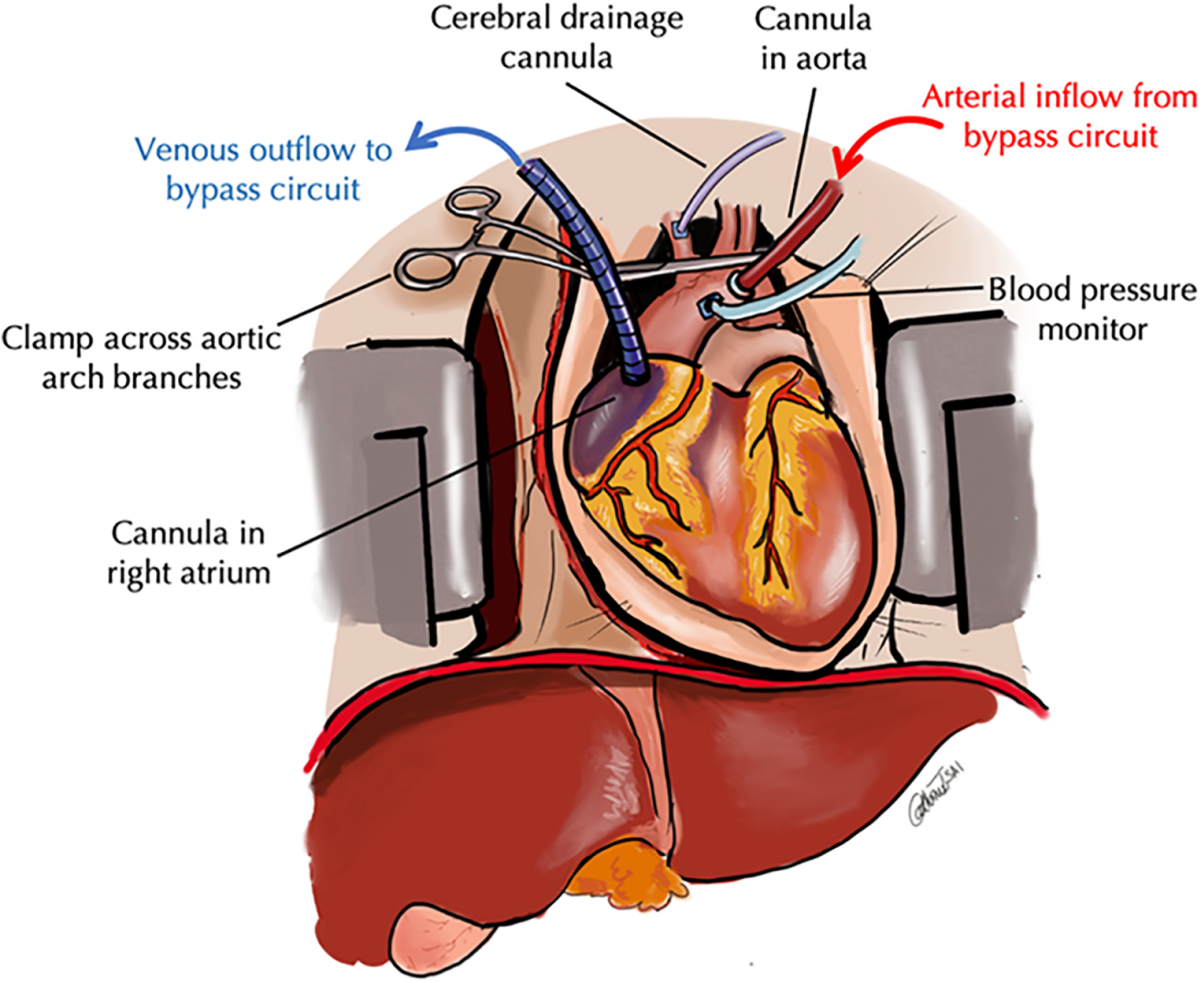
Schematic of thoracoabdominal normothermic regional perfusion. In TA-NRP, the standarad DCD pathway is followed. After declaration of cardiopulmonary arrest and the mandatory hands off period, a rapid midline sternotomy is performed and the aortic arch vessels are ligated. A venous outflow cannula is placed in the right atrium and an arterial inflow cannula is placed in the aortic arch and CPB is inititated. An additional cannula is placed is the innominate artery to vent any collateral cerebral flow and an aortic root cannula is placed to meaure blood pressure to ensure adequate perfusion while on CPB.

For TA-NRP, access was via a midline sternotomy followed by ligation of the aortic arch vessels. Cannulas were placed in the right atrium and ascending aorta. A cannulae was placed in the innominate artery to vent collateral cerebral flow (Figure 2). TA-NRP was initiated with a modified CPB circuit. After cannulation, the donor was re-intubated and maintained on mechanical ventilation. Arterial pressures were obtained via an aortic root cannula. Serial gases and lactates were drawn every 5 min and circuit parameters adjusted accordingly. Flow was targeted to maintain a rate of 4–5 L/min with a mean arterial pressure (MAP) of 60 and temperature above 37°C. Vasoactive medications were administered via the CPB circuit.

In all cases, WLST and declaration of death was in accordance with local organ procurement organization (OPO) standards. WLST occurred in either the operating room, peri-operative recovery areas or the ICU as per local OPO and hospital guidelines. Heparin administration for TA-NRP was 50,000 units and timing of administration was unchanged from standard DCD recovery methods. Organ allocation was conducted in accordance with UNOS allocation guidelines.

For pooled data analysis, fWIT was defined as when the donor's systolic blood pressure (SBP) was <80 mmHg and/or the oxygen saturation was <80%, including the hands-off period mandated by OPO policy, and time until initiation of TA-NRP bypass. Time from extubation to initiation of TA-NRP was defined as tWIT (2, 20). Center-specific acceptance criteria for DCD liver allografts was at the discretion of each transplant center. CIT was considered from aortic crossclamp in the donor to out of ice time in the recipient. One donor was placed on an *ex vivo* normothermic perfusion pump; CIT for this donor was considered from aortic crossclamp in the donor to placement on pump.

## Results

Of the 30 donors that underwent TA-NRP with the intent for thoracic and/or abdominal DCD procurement, 27 livers were successfully allocated at the time of procurement. Of these 27 livers, 7 livers allografts were declined for exceeding fWIT by the accepting center, 2 were declined for anatomy/biopsy, 1 was declined for recipient issues post-crossclamp (reallocated but ultimately discarded, Figure 1). Within the cohort of liver allografts successfully transplanted, there was one case of successful liver re-allocation after decline by the primary center; the difference was the acceptable fWIT by each accepting center.

**Figure 3:**
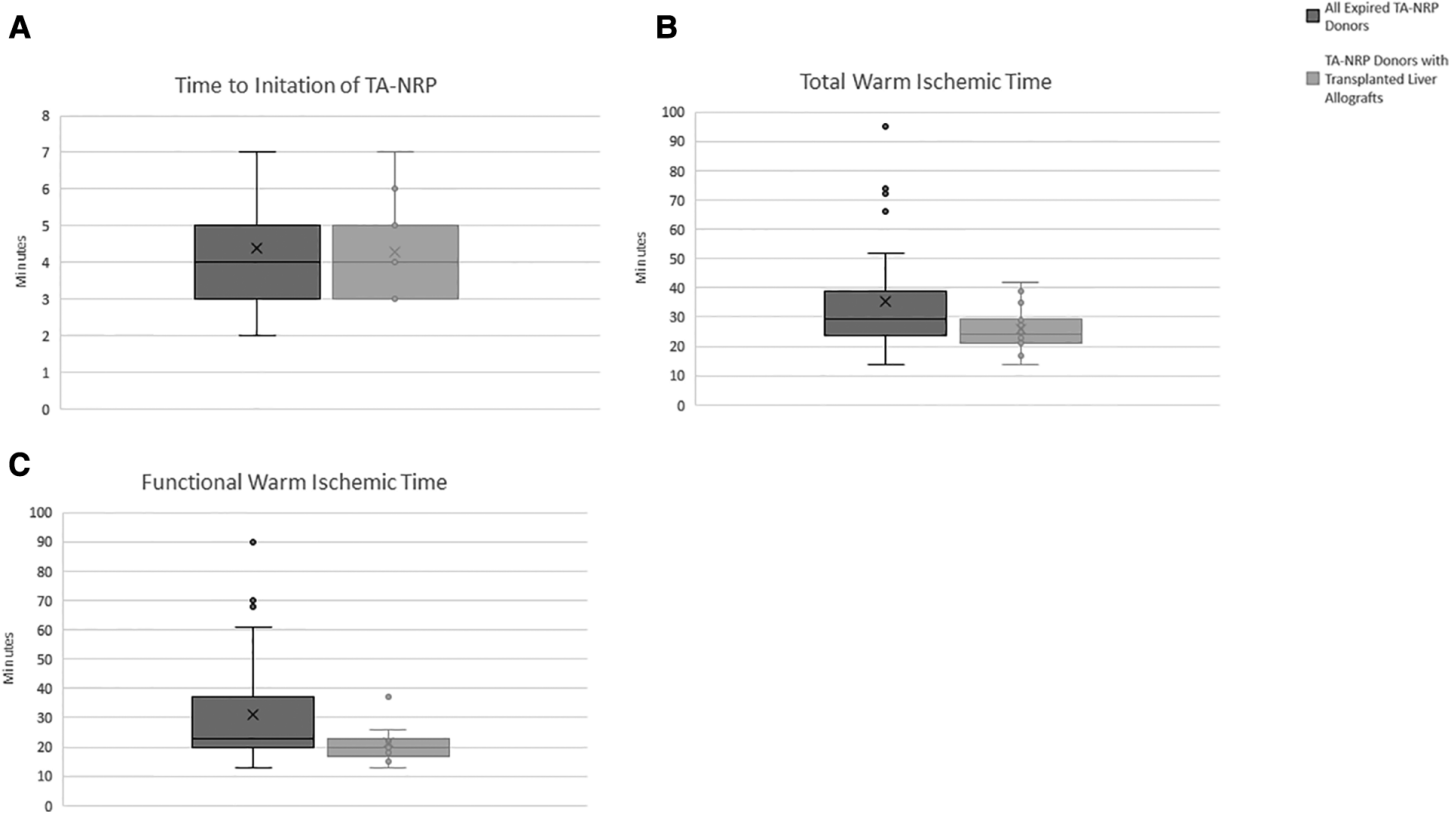
Donation after circulatory death variables. (**A**) Time to intiation of TA-NRP. (**B**) Total Warm Ischemic Time. (**C**) Functional Warm Ischemic Time. Dark gray bars represent all expired TA-NRP donors over the study perdios. Light gray bars represent TA-NRP donors that expired and the liver allograft was transplanted. “X” represents the median value with the whiskers demonstrating the interquartile range. Total warm ischemic time is defined from extubation to intiation of TA-NRP. Functional warm ischemic time is defined as systolic blood pressure less than 80 mmHg or oxygen saturation less than 80% until intiation of TA-NRP.

In all 30 cases of TA-NRP, there were no technical issues with cannulation that prevented successful initiation of TA-NRP (i.e., aortic dissection, failure to cannulate). Figures 3A–C details the time to initiation of TA-NRP from first declaration of death, fWIT and tWIT for all 30 DCD donors that expired and for the 16 livers recipient-donor pairs included in the study. Median time to cannulate from completion of the hands off period to start of TA-NRP was 4 min (3–5 min).

In total, 17 liver allografts were transplanted following TA-NRP DCD procurement at 9 centers; 8 centers were reached for inclusion in the study for a total of 16 allografts. TA-NRP was conducted in 14 DCD donors to assess and procure thoracic and abdominal organs for transplant. In two additional cases, TA-NRP was conducted in DCD donors for the procurement of abdominal organs only. In 93.8% of cases, liver allografts were placed with a local transplant center within the OPO's donation service area; in one case the liver was placed outside the OPO's donation service area but with the same region.

Of the 16 donor-recipient pairs included, the median donor age was 32 years old (25.8–43.3 years), with the oldest donor being 61 years old, and the median body mass index (BMI) was 27.4 (23.9–28.9) (Table 2). Donors had no major medical co-morbid conditions; most (87.5%) were male. Table 1 details the donor median peak and terminal liver function laboratory values. The median fWIT was 20.5 min (17.5–23.0 min); two allografts were transplanted with a fWIT of 37 min (Figure 3C). Median time on TA-NRP CPB was 66.0 min (54–74.5 min). The median starting and terminal lactates were 8.3 mmol/L (7.4–8.7) and 5.2 mmol/L (4.7–6.6), respectively with a median starting base deficit of −6.5 mEq/L (−7.8 to −2.5) and terminal base excess of 1.0 mEq/L (0–5.5). The UK DCD risk score was calculated for each donor-recipient pair: 8 low risk, 7 high risk, and 1 futile.

**Table 1:** Donor characteristics.

COD (*n*, *n* = 16)	
Hanging Anoxia Blunt Trauma GSW Stroke	2 5 7 1 1
Age (years)	32 (25.8–43.3)
Sex (*n*, % male)	14, 87.5%
BMI	27.4 (23.9–28.9)
AST (U/L)	
Peak Terminal	84.5 (49.8–149.5) 36 (23–53.3)
ALT (U/L)	
Peak Terminal	91.0 (34.8–167.0) 40 (24.5–74.3)
TB (U/L)	
Peak Terminal	1.0 (0.8–1.1) 0.8 (0.5–0.9)
tWIT (min)	24.5 (21.8–29.3)
fWIT (min)	20.5 (17.5–23.0)
Lactate (mmol/L)	
Initial Terminal	8.3 (7.4–8.7) 5.2 (4.7–6.6)
Pump time (min)	66.0 (54.0–74.5)

Data presented as median with interquartile range (IQR) Q1–Q3, unless otherwise specified. COD, cause of death; GSW, gun shot wound; MVC, motor vehicle collision; BMI, body mass index; AST, aspartate aminotransferase; ALT, alanine transaminase; TB, total bilirubin; tWIT, total warm ischemia time; fWIT, functional warm ischemia time.

**Table 2:** Recipient characterisatics and transplant course.

Recipient	Etiology of Liver Disease	Age	Sex	Lab MELD	Listed MELD	Prior Abdominal Surgery (Y/N)	Re-Transplant (Y/N)	CIT (m)	PNF (Y/N)	Peak AST (U/L)	Peak ALT (U/L)	Peak Lactate (mmol/L)	ICU LOS (days)	Post-Op Hospital LOS (days)	Length of Follow Up (days)	Transplant Related Complications and Readmissions
1	HCV, HCC	60	M	10	27	*N*	*N*	511	*N*	2,239	2,131	7.8	1	5	270	Anastomotic biliary stricture requiring readmission, ERCP and stent; developed recurrent diffuse HCC, passed at day 270
2	HCV, Alcohol, HCC	59	M	7	27	*N*	*N*	500	*N*	3,420	1,735	5.2	7	10	366	Readmission for abdominal pain, normal allograft function, no intervention
3	A1AT, NASH	51	M	24	33	*N*	*N*	500	*N*	286	139	3.5	1	17	367	*N*
4	NASH	42	M	21	19	*N*	*N*	470	*N*	3,220	1,960	4.9	5	9	333	*N*
5	Alcohol	69	M	22	22	*N*	*N*	210	*N*	317	494	0.9	2	7	326	*N*
6	Intrahepatic cholangiocarcinoma (RESTORE Trial)	59	M	8	7	Y, left liver lobectomy	*N*	342^a^	*N*	6,298	2,072	3.4	2	7	264	*N*
7	NASH/hemochromatosis	66	F	27	27	Y, laparoscopic cholecystectomy and hysterectomy	*N*	288	*N*	1,170	920	1.7	1	4	259	*N*
8	AIH	49	M	17	17	*N*	*N*	310	*N*	376	311	2.8	4	7	258	Readmission for SOB, normal allograft function
9	Alcohol	50	M	26	26	*N*	*N*	226	*N*	318	155	1.8	1	5	257	Anastomotic biliary stricture requiring outpatient ERCP and stent
10	Alcohol	67	M	24	24	*N*	*N*	200	*N*	720	421	1	3	7	234	*N*
11	Alcohol	66	M	19	17	*N*	*N*	218	*N*	431	350	2	1	9	606	*N*
12	HCV, HCC	59	M	19	22	*N*	*N*	296	*N*	1,412	1,580	1.8	2	7	580	*N*
13	Alcohol	52	M	18	21	*N*	*N*	205	*N*	814	783	5.5	1	5	548	*N*
14	Alcohol	48	M	22	22	*N*	*N*	204	*N*	1,291	1,540	2.5	2	7	533	*N*
15	Alcohol	35	M	32	32	*N*	*N*	313	*N*	642	444	3.4	5	7	154	Re-operation for anastomic bleed on POD2
16	Alcohol	63	M	18	17	*N*	*N*	210	*N*	923	614	3.1	1	8	503	Anastomotic stricture, ERCP and stent

^a^ Allograft #6 was additionally placed on an ex-vivo normothermic perfusion pump prior to implantation.

Recipient characteristics and transplant outcomes for each recipient are detailed in Table 2. Summative data of the study cohort are described in Table 3. Median length of follow up was 329 days, or approximately 11 months (259–510 days, or 8.6–17 months). Median recipient age was 59 years; 93.8% were male. Median listed MELD and MELD at time of transplant were 22 and 21, respectively. Two recipients had prior abdominal surgery; none of the recipients were undergoing re-transplantation. Median allograft CIT was 292 min (210.0–374.0 min; 4.9 h, 3.5–6.2 h). Of note, allograft #6 was additionally placed on an *ex vivo* normothermic perfusion pump prior to implantation. No allografts demonstrated PNF. Median peak liver enzymes and lactates following transplant were as follows: AST 923 IU/L (536.5–1825.5), ALT 783 IU/L (432.5–1657.5) and lactate 3.1 (1.8–4.2). Three recipients meet EAD criteria by Olthoff by AST levels. Median ICU and hospital LOS were 2 and 7 days, respectively.

**Table 3:** Summative recipient data.

Age (years)	59 (50.5–64.5)
Sex (*n*, % male)	15, 93.8%
MELD	
Listing At transplant	22 (20–27) 21 (18–24)
CIT (min, h)	292 min (210–374) 4.9 h (3.5–6.2)
ICU LOS (days)	2 (1–2.5)
Hospital LOS (days)	7 (6.8–9)
Peak AST (U/L)	923 (536.5–1,825.5)
Peak ALT (U/L)	783 (432.5–1,657.5)
Peak Lactate (mmol/L)	3.1 (1.8–4.2)

Data presented as median with interquartile range (IQR) Q1–Q3, unless otherwise specified. MELD, model of end stage liver disease; CIT, cold ischemic time; LOS, length of stay; AST, aspartate aminotransferase; ALT, alanine transaminase.

No intra-operative deaths occurred. One patient underwent re-operation on post-operative day 2 for an anastomic bleed. There were no re-operations due to vascular thrombosis or biliary complications, and no patient has required re-transplantation. Three recipients required management of biliary strictures; one during the index admission and two in subsequent encounters. One recipient was re-admitted for an anastomotic biliary stricture necessitating endoscopic retrograde cholangiopancreatography (ERCP) and stenting on post-operative day 17. Subsequent ERCP at 4 months post-operatively showed no residual stricture; the stent was removed, and the patient has had no recurrent biliary complications; this recipient did expire secondary to hepatocellular carcinoma (HCC) recurrence at 9 months post-transplant. The second recipient underwent outpatient ERCP with stent placement for an anastomotic biliary stricture on post-operative day 15. Stents were removed at 2 months with no residual stricture at 8.5 months post-operatively. The third patient developed an early anastomotic stricture on post-operative day 5; the patient underwent repeat ERCP at 2.5 months post-operatively with residual anastomotic stricture requiring additional stent exchanges. Stents were removed on post-operative day 377 with no residual extrahepatic or intrahepatic stricture on ERCP; the patient has had no residual biliary complications at 503 days post-transplant. Clinically, no patient developed IC or required evaluation for concern for IC with ERCP, MRCP or PTC. Other than the patient that expired secondary to recurrent HCC, there was no graft or patient loss at last follow up.

## Discussion

The data from our retrospective multi-center series of liver transplants after DCD donation using TA-NRP in the US demonstate excellent early allograft and patient outcomes with no evidence of PNF or IC in our 16 patient cohort. These data are in line with a recent publication by Sellers et al., and expand the available US literature supporting TA-NRP as a viable recovery technique that can expand DCD organ utilization with reassuring outcomes (18). Moreover, these data are also in line with abdominal and TA-NRP liver allograft outcomes described in Spain, Italy and the UK which demonstrate reduced rates of PNF, EAD and IC (6, 8, 10–12, 21–23).

There are limitations to our retrospective, observational study, including the small sample size. It is also important to note that most donors were young, with low BMI and fWIT ≤30 min. These donor characteristics are consistent with prior publications that suggest shorter fWIT and younger donors prognosticate better outcomes after DCD with super rapid recovery (SRR) (2, 3, 19). Althougth our median follow up was just under 1 year, all recipients in our cohort were at greater than 3 months post-transplant at the time of publication. In a recent multi-center study evaluating IC in a large cohort of US DCD liver allografts after SRR, Croome et al. reported that IC occurred at a median of 1 month after transplant with the majority of cases diagnosed by 3 months post-transplant (24). Together, these data support that TA-NRP may be a promising modality to limit IC in DCD donors.

In consideration of donor age and NRP, in Savier's et al. retrospective study comparing abdominal NRP DCD to DBD donors, inclusion criteria for DCD donors was age less than 66 while DCD donors in a similar study by Ruiz et al. averaged 62 years old (6, 7). These studies found similar rates of biliary complications and graft survival as compared to DBD donors. Comparing abdominal NRP to SRR for DCD donors, Hessheimer et al. found improvement in overall biliary complications and graft survival utlizing abdominal NRP with a median age above 50 in both groups (11). Two donors were ≥50 years of age in our study cohort; neither developed early allograft dysfunction, IC or PNF. While further data on the US experience with TA-NRP needs to be examined, our early data suggest that NRP allows for safe expansion of DCD donor criteria, such as age, with good outcomes. As transplant programs consider how to utilize TA-NRP as a recovery modality, granular data will help centers comfortably expand acceptance criteria of uncommonly utilized DCD allografts (i.e., older donors, macrosteatotic allografts).

Additional studies are needed to determine the optimal duration of TA-NRP for favorable abdominal allograft outcomes. In contrast with abdominal NRP with supraceliac aortic occlusion, TA-NRP offers the ability to wean off bypass and sustain autologus perfusion with the donor heart. Alternatively, if heart recovery for transplant is not intended, the need to wean the circuit for assessment of the heart is unnecessary and the donor can be maintained on the CPB circuit. The benefits of remaining on bypass compared to autologous perfusion by the donor heart in the context of abdominal organ allograft recovery are unknown. In 14 of the 16 liver allograft donors, total time on bypass was determined by the cardiothoracic team. In the 2 cases of TA-NRP for abdominal organ evaluation and procurement only, a minimum bypass time of 60 min was used based on prior abdominal NRP studies (6, 9, 11). Preclinical studies evaluating kidney allograft function after NRP found improved function and a decrease in markers of tissue injury after 4 h of NRP compared to no NRP (25). Liver allografts in porcine models have shown benefits with as little as 30 min of NRP (26). Liver allograft data from clinical studies suggests 2 h of abdominal NRP is associated with a reduction in lactate and graft outcomes equivalent to DBD donors (6). Based on our experience with TA-NRP, 60 min of perfusion of abdominal organs provided excellent short-term outcomes. As the abdominal transplant community's experience with TA-NRP increases, ongoing research efforts can provide data to help tailor the duration of perfusion to optimize abdominal allografts.

Currently, there is no uniform definition of fWIT across centers and transplant specialty teams. For example, at UCSD, our cardiothoracic team routinely used a MAP <50 mmHg and/or an oxygen saturation <70% to define the start of fWIT. In comparison, our abdominal transplant team defines fWIT as SBP <80 mmHg or oxygen saturation <80%. Additionally, across liver transplant centers, there is variation in definition of acceptable fWIT; i.e., while one center declined a liver for tWIT of >20 min, another center accepted this liver with a fWIT of ≤30 min. In their recent single center report of TA-NRP on 3 liver allografts, Merani et al. utilized a SBP of <50 mmHg to define fWIT (20). This is a similar definition of fWIT used in the European abdominal NRP studies with short- and long-term results comparable to DBD donors (6, 7). In all these studies, while the definition of initiation fWIT was more liberal, fWIT >30 min was considered preclusive to transplant. Generally, most transplant organizations concur that a fWIT of ≤30 min for DCD liver allograft donors is recommended, but the definitition of initiation of an acceptable fWIT remains variable. Most recently, the International Liver Transplantation Society generated a consensus statement recommending fWIT be defined as when either oxygen saturation is <80% or the MAP is <60 mm Hg (2). Utilizing a consensus definition of fWIT would standardized communication across centers and improve the ability to critically analyze outcomes of DCD donors.

During fWIT, hypoxemic ischemia incites cellular injury effectuated by free radicals, activated immune cells and vascular stasis that continues until rapid cooling with preservation fluid occurs. With NRP, this warm ischemic time prior to initiation of bypass may offer the benefits of ischemic preconditioning (25, 26). Once on bypass, NRP has been postulated to restore cellular substrates that are depleted during the window of warm ischemic injury and potentially allow recovery of donor allografts (25–28). Several mechanisms and biomarkers have been implicated in the benefits of ischemic preconditioning, though their precise roles remain unknown (29). In preclinical studies, hypoxia inducible factor-1α and nitric oxide pathways are upregulated early after initiation of NRP (25). Adenosine, xanthine and superoxide dismutase are also implicated in playing protective roles in liver and kidney allografts after NRP in animal models (26–28, 30). In existing clinical studies, routine liver function studies have demonstrated that an ALT <3× the upper limit of normal at the initiation of NRP and an ALT <4× the upper limit of normal at the end of NRP could help evaluate graft viability (7, 12). A more recent retrospective analysis of the Spain experience suggested transaminases <200 IU/L with a downtrending lactate implies adequate graft viability in conjunction with other standard measures (8). Reduction in lactate levels are likely to be of prognostic benefit, similar to the *ex vivo* perfusion pump, though their levels are also affected by non-perfused tissues in the donor (i.e., the brain) (22). Understanding this interplay between ischemic preconditioning, allograft donor recovery and allograft outcomes may highlight fundamental mechanisms and point of care biomarkers to assess allograft usability.

Despite the relative success with abdominal NRP in other countries, routine use of NRP has not taken hold in the US. Limitations include availability of a qualified perfusion team to run a perfusion circuit, as well as surgeon experience with femoral, abdominal or thoracic cannulation and management of the perfusion circuit. Additional considerations that have limited successful implementation of NRP include pre-mortem procedures, such as femoral arterial and venous sheath placement; a benefit of TA-NRP is that there are no pre-mortem cannulation considerations. As TA-NRP for cardiac donation continues to grow in the US, there is also the opportunity to learn from our thoracic colleagues and expand routine recovery techniques to include TA-NRP for abdominal only recovery. Moreover, *ex situ* NMP and *ex situ* HMP offer alterative modalities that allow for metabolic recovery of DCD allografts with improved outcomes as compared to static cold storage (15–17, 31). While ongoing evaluation of these technologies and their impact on DCD liver allografts is needed, Mohkam et al. did demonstrate similar rates of EAD, non-anastomotic biliary stricture and graft loss when comparing *in situ* NRP and *ex situ* NMP (32). *In situ* NRP does have the ability to simultaneously impact multiple poterntial allografts as compared to the need for multiple *ex situ* devices, and has been shown to demonstrate improved organ utilization for DCD donors in the UK (33). Importantly, these technologies are not mutually exclusive and have great potential to improve the current US DCD transplantation landscape, which has historically utilized DCD organs at rates far lower than in Europe. While there has been a positive impact on organ utilization with increasing NRP use in the UK, the margin for improvement is much greater in the US and remains to be determined.

Moving forward, creating standardized guidelines early in our TA-NRP experience will help the transplant community better understand and optimize liver allografts from DCD donors after TA-NRP. Development of a granular data registry for allografts from NRP DCD donors will help assess short- and long-term outcomes. Addiontional avenues of research including impact of NRP on donors families, recipient quality of life measures, and determination of how NRP effects evaluation of early allograft failure by prior scoring systems should all be evaluated. Together, these efforts will aid our ability to continually consider and readdress how we approach DCD donors while increasing access to suitable organs for transplant and improving transplant outcomes.

## Data Availability

The original contributions presented in the study are included in the article/**Supplementary Material**, further inquiries can be directed to the corresponding author.
